# Microbiome-metabolome dysbiosis of bronchoalveolar lavage fluid of lung cancer patients

**DOI:** 10.3389/fmicb.2025.1669172

**Published:** 2025-11-12

**Authors:** Jing Wang, Wei Su, Qin Chen, Jiemin Zhou, Xinyue Wang, Richeng Jiang, Junling Li, Puyuan Xing

**Affiliations:** 1 Department of Medical Oncology, Beijing Chaoyang Sanhuan Cancer Hospital, Beijing, China; 2 Department of Endoscopy, Tianjin Medical University Cancer Institute and Hospital, National Clinical Research Center for Cancer, China Key Laboratory of Cancer Prevention and Therapy, Tianjin’s Clinical Research Center for Cancer, Tianjin, China; 3 Department of Respiratory and Critical Medicine, Tianjin Chest Hospital, Affiliated Chest Hospital of Tianjin University, Tianjin, China; 4 Vision Medicals Center for Infectious Diseases, Guangzhou, China; 5 Center for Precision Cancer Medicine & Translational Research, National Clinical Research Center for Cancer, Key Laboratory of Cancer Prevention and Therapy, Tianjin's Clinical Research Center for Cancer, Tianjin Medical University Cancer Institute & Hospital, Tianjin, China; 6 Department of Medical Oncology, National Cancer Center, National Clinical Research Center for Cancer, Cancer Hospital, Chinese Academy of Medical Sciences and Peking Union Medical College, Beijing, China

**Keywords:** microbiome, metabolomics, lung cancer, diagnosis, BALF

## Abstract

**Background:**

Recent studies indicate that microorganisms significantly influence lung cancer pathogenesis. This research explores the variations in microbiota and metabolites in the lower respiratory tract between lung cancer patients and individuals with benign pulmonary lesions to identify potential diagnostic biomarkers.

**Methods:**

Two hundred eight patients undergoing bronchoscopy at Tianjin Cancer Institute & Hospital and Tianjin Chest Hospital from October 2022 to October 2023 were screened. Ninety-five bronchoalveolar lavage fluid (BALF) was collected for metagenomic sequencing and untargeted metabolomic analysis. Comparisons of microbial diversity, taxonomic composition, and metabolite profiles were conducted between groups with lung cancer and benign lung conditions.

**Results:**

The cohort comprised 70 patients with lung cancer and 25 with benign lung lesions. Patients with lung cancer showed significantly reduced *β*-diversity (*p* = 0.005). Predominant microbes in lung cancer cases included *Streptococcus*, *Haemophilus influenzae*, and *Veillonella parvula*. A microbial-based diagnostic model differentiated lung cancer from benign lesions with an AUC of 0.931 (95%CI: 0.916–0.946). Metabolites increased in lung cancer were Citric acid, N-Acetylneuraminic acid, Oxoglutaric acid, and Neopterin, whereas L-Tryptophan, Uridine, 3-Hydroxybutyric acid decreased. The KEGG pathways suggest a significant link between microbial presence and both tumorigenesis and progression.

**Conclusion:**

Specific microbial patterns in the lower respiratory tract of lung cancer patients could assist in the auxiliary diagnosis of the disease. The notably altered microorganisms and metabolites in the BALF from lung cancer patients, as opposed to those with benign conditions, correlate with cancer initiation and advancement.

## Introduction

According to the 2023 American Cancer Report, lung cancer remains the second most common malignant tumor and the leading cause of cancer deaths worldwide (Jemal et al., 2003; Leiter et al., 2023). In China, lung cancer not only leads in cancer-related mortality but also shows the most rapid increase in incidence over the past three decades (Oncology Society of Chinese Medical Association, 2024). Approximately half of the lung cancer patients are diagnosed at an advanced stage, with an overall five-year survival rate of only 21.7% (Ettinger et al., 2022), and a mere 8% for those with advanced disease (Leiter et al., 2023). This situation underscores the critical need for early lung cancer diagnosis and new therapeutic approaches.

In recent years, advances in microbiome technology have propelled the study of the relationship between human microbiota and diseases to the forefront of life sciences. From the pivotal role of gut microbes in metabolic diseases and colorectal cancer to the involvement of skin, oral, and respiratory microbiota in local immune regulation, the microbiome has been established as a key regulator of host physiological and pathological processes (Heintz-Buschart and Wilmes, 2018). The respiratory microbiome, once thought to be sterile (Dickson et al., 2016), has gained attention, particularly its potential link with lung cancer pathogenesis and new therapeutic targets. Technological advances, such as 16s rRNA and metagenomics, have revealed a diverse microbial presence in the lungs, including bacteria, fungi, and viruses, albeit in low abundance. Studies have documented an increase in microbes such as *Veillonella*, *Streptococcus* in the saliva/sputum of lung cancer patients (Baranova et al., 2022; Vogtmann et al., 2022; Roy et al., 2022; Druzhinin et al., 2021; Hosgood et al., 2021). While the distinction between lung and respiratory tract microbiota is often unclear, and though oropharyngeal and upper respiratory microbiota related to lung cancer are heavily influenced by environmental factors (Mendez et al., 2019). Huang demonstrated that the microbiota in BALF closely resembles that in lung tissue (Huang et al., 2019). In lung cancer patients, *Streptococcus*, *Neisseria*, *Haemophilus influenzae* are prevalent in BALF (Liu et al., 2018; Jin et al., 2019). Tsay found that *Streptococcus* and *Veillonella* are significantly enriched in the lower airways of lung cancer patients and enhance tumor proliferation, invasion, and infiltration via the ERK and PI3K signaling pathways (Tsay et al., 2021). Despite numerous studies exploring the correlation between microorganisms and lung cancer, inconsistencies in sample sources and conclusions, such as Liu provided evidence that streptococci are abundant in the microbiota associated with lung cancer and staphylococci are almost absent, suggesting the pro-carcinogenic effect of streptococcus and the protective effect of staphylococcus in the development of lung cancer (Liu et al., 2018). This hypothesis contradicts another study confirming that staphylococci induce DNA damage during carcinogenesis, and streptococci may play a role in its prevention (Urbaniak et al., 2016). The main reason for this is the small sample size of the existing studies, which highlights the need for further research to provide more conclusive evidence on the role of microorganisms in lung cancer development.

Currently, the precise mechanisms through which microbiota promote the onset and progression of malignant tumors are not well understood. Microbial metabolites are key mediators of interactions between the microbiota and the host, influencing the development and progression of lung cancer by regulating signaling pathways, epigenetic modifications, and immune responses. These metabolites represent a significant area for investigating how microbiota contribute to cancer progression. Short-chain fatty acids (SCFAs), produced by specific gut bacteria, are crucial in maintaining intestinal immune homeostasis. SCFAs modulate various signaling pathways, including inhibiting tumor growth by promoting Wnt-mediated differentiation and reducing proliferation via the Wnt signaling pathway (Feitelson et al., 2023). Wang demonstrated that *Clostridiales* were more prevalent in the immune-activated subtype of triple-negative breast cancer. Clinically, the microbe-derived metabolite trimethylamine N-oxide (TMAO) has been linked to enhanced immunotherapy efficacy. TMAO activates the endoplasmic reticulum (ER) stress kinase PERK, triggers gasdermin E-mediated tumor cell pyroptosis, and boosts CD8 + T cell-mediated anti-tumor immunity *in vivo* in triple-negative breast cancer (Wang et al., 2022). Research in microbial metabolomics remains primarily focused on gut microbiota, with extensive application in studies on inflammatory lung diseases such as asthma, respiratory distress syndrome, and cystic fibrosis. However, its association with lung cancer is less explored. A pioneering study from Spain investigated metabolic alterations in BALF, identifying significant differences between lung cancer patients and healthy controls, with glycerol and phosphate emerging as potential sensitive and specific biomarkers for lung cancer diagnosis and prognosis (Callejón-Leblic et al., 2016). Additionally, multiple microbiota in the lower respiratory tract beyond neoplastic lesions can influence the local ecological environment through SCFAs (Jin et al., 2018; Yue et al., 2020).

In light of this background, BALF was collected from patients with lung cancer and benign lung diseases for a combined analysis of microorganisms and metabolites. This study aims to identify distinct differences in the respiratory microbiome composition between lung cancer patients and those with benign lung diseases, and to elucidate how the microbiome influences lung cancer development, validate the clinical utility of specific microbial biomarkers in lung cancer diagnosis.

## Participants and methods

### Patients

**Figure 1:**
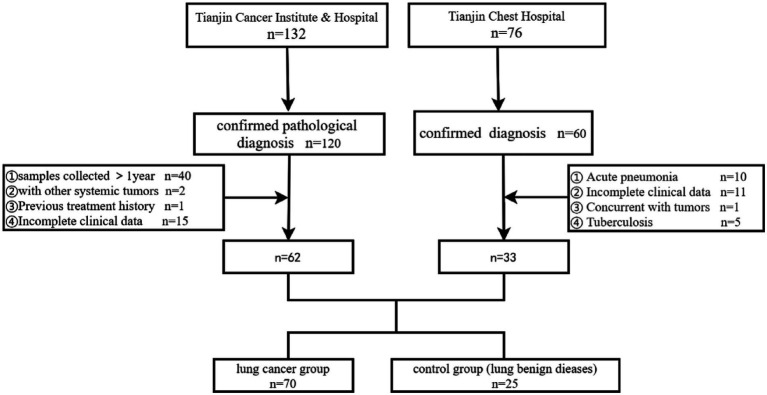
Case screening process.

Patients who visited Tianjin Cancer Institute & Hospital and Tianjin Chest Hospital for the first time from October 2022 to October 2023 and underwent bronchoscopy were screened. The final diagnosis relied on pathological and imaging findings, and the study cohort was selected based on inclusion and exclusion criteria. This study received approval from the Ethics Committee of Tianjin Cancer Institute & Hospital (E20220909). The control group comprised patients with benign lung diseases, excluding lung cancer. Exclusion criteria for the control group included: (1) Age under 18 years; (2) Use of antibiotics, corticosteroids, cytokines (such as interleukins, interferons), methotrexate, immunosuppressive cytotoxic drugs, or high-dose probiotic supplements within 3 months prior to bronchoscopy (except for high-dose probiotic health products (<10^9 CFU/d), which were excluded if used within 3 days); (3) Acute pneumonia, pulmonary tuberculosis, AIDS, diabetes, history of lung surgery, malignancies in the lungs or other organs; (4) Any condition deemed unsuitable for bronchoscopy. Inclusion criteria for lung cancer patients were: (1) Ages 18–85, with no contraindications for bronchoscopy; (2) No previous bronchoscopy; (3) Complete clinical and pathological data; (4) At least one measurable lesion according to the Response Evaluation Criteria in Solid Tumors version 1.1 (RECIST 1.1). Exclusion criteria for lung cancer patients were: (1) Under 18 years of age; (2) Concurrent conditions such as pneumonia, bronchial asthma, tuberculosis, chronic bronchitis, interstitial lung disease, chronic obstructive pulmonary disease (COPD), AIDS, diabetes, history of lung surgery, or malignancies in other systems; (3) Use of antibiotics, corticosteroids, cytokines (such as interleukin, interferon), methotrexate, immunosuppressive cytotoxic drugs, and high-dose probiotic health products within 3 months prior to bronchoscopy (with an exception for high-dose probiotic health products used within 3 days); (4) Incomplete clinical information (The process for screening cases is illustrated in Figure 1).

### Sample collection

The bronchoscopy is conducted using an OLYMPUS BF-20 fiberoptic bronchoscope (FB). When the bronchoscope reaches the lesion site, the FB is wedged into the segmental bronchial opening at or near the lesion. Sterile saline (0.9%) at room temperature is used, administered in 20 ml aliquots for a total volume of at least 80 ml, followed by suction at a negative pressure of 13.3 kPa. A recovery rate of over 40% is required for all BALF samples. The BALF is filtered through a double layer of gauze; 2 ml is transferred into a sterile 5 ml cryovial for metabolite detection and the remainder into a 15 ml sterile plastic tube for microbial detection. Tube openings are immediately sealed with parafilm and samples are stored at −80 °C within 30 min of collection by specially trained personnel.

### Metagenomic sequencing

Samples for microbial detection are transported under a complete cold chain at −80 °C to Guangdong Vision Medical Technology Company for DNA extraction and analysis. Host-derived DNA is removed using 1 U of Thermo Fisher nuclease and 0.5% Tween 20 from SIGMA. Microbial DNA extraction is performed using the QIAGEN QIAamp® UCP Pathogen DNA Kit, following the manufacturer’s protocol. cDNA transcription is performed using reverse transcriptase and dNTPs from Thermo Fisher. Library construction for DNA and cDNA samples is conducted using the Illumina Nextera XT DNA Library Prep Kit. Library quality is assessed using the AGILENT Qubit dsDNA HS Assay Kit on the Agilent 2,100 Bioanalyzer with the Agilent High Sensitivity DNA Kit. Libraries are quantified accurately using Q-PCR to ensure quality, based on insert size and effective concentration. Metagenomic sequencing depth was 20 million reads. We performed quality control on the sequenced raw reads using fastp (version 0.23.4) to filter out low-quality data (filtering criteria are described below), resulting in clean reads. The read filtering steps were as follows: (1) removal of reads containing adapters; (2) removal of reads with an N content exceeding 10%; (3) removal of low-quality reads (where bases with a quality value≤20 account for more than 50% of the entire read). Detailed quality control information has been added to the attachment (Supplementary Table 1).

### Non-targeted metabolomics process

The experiment utilizes a Waters UPLC I-Class Plus system coupled with a Q Exactive high-resolution mass spectrometer (Thermo Fisher Scientific, USA) for metabolite separation and detection. Raw mass spectrometry data are imported into Compound Discoverer 3.3 (Thermo Fisher Scientific, USA) software, which integrates the BMDB (BGI Metabolome Database), mzCloud database, and ChemSpider online database for analysis. A data matrix containing metabolite peak areas and identification results is generated and processed for further analysis. Software information: Compound Discoverer Version: v.3.3. Parameters: Parent ion mass deviation: <5 ppm Mass deviation of fragment ions: <10 ppm, Retention time deviation: < 0.2 min. Official Website: https://mycompounddiscoverer.com/.

### Bioinformatics analysis

#### Microbial bioinformatics analysis

After obtaining raw sequencing data, quality control was conducted using fastp. The quality-controlled data were aligned to the host reference genome sequence GRCh38 to remove host-derived sequences. The filtered sequences were then aligned to the Metaphlan/Kraken2 (version 2.1.3) database to ascertain species abundance for further analysis. Species *α*-diversity was assessed using indices such as Richness, ACE, Chao1, Shannon, Simpson, and Evenness. *β*-diversity was analyzed using Principal Coordinates Analysis (PCoA) and Non-metric Multidimensional Scaling (NMDS) based on the Bray-Curtis distance. Statistical significance of β-diversity differences was determined using Permutational Multivariate Analysis of Variance (PERMANOVA). To identify potential biomarkers, LEfSe (LDA Effect Size) analysis was utilized. Differences in species abundance between groups were tested using the Wilcoxon test or K-S test to generate *p*-values. Species showing significant differences were selected based on p-values and LDA scores, and relevant plots were produced. Using the Sequential Forward Feature Selection algorithm and Random Forest Classifier, a subset of species that clearly differentiated the groups was selected to construct a diagnostic model. Model predictive performance was evaluated based on the area under the receiver operating characteristic curve (AUC). This project employed 10-fold cross-validation to evaluate model performance.

HUMAnN2 (version 0.11.1) was employed for gene function annotation and analysis. This tool analyzes microbial pathway abundance from metagenomic and metatranscriptomic data using the MetaPhlAn and ChocoPhlAn pan-genome databases, covering species such as archaea, bacteria, eukaryotes, and viruses. HUMAnN2 offers results at the genome, gene, and pathway levels, using the UniRef 9 database for gene family definitions, MetaCyc 10 for pathway definitions, and MinPath for minimal pathway sets. Bowtie 11 and Diamond 12 were utilized to enhance nucleic acid and protein-level searches, respectively. Post-analysis, HUMAnN2 provided MetaCyc pathway results and gene family annotations, which were then mapped to GO and KO annotation results.

#### Metabolomics bioinformatics analysis

Data exported from Compound Discoverer were imported into metaX for preprocessing and further analysis. Metabolites were identified by referencing the Human Metabolome Database (HMDB), and pathway annotations were performed using the KEGG PATHWAY database to elucidate major biochemical metabolic pathways and signal transduction pathways involved. Differential analysis between comparison groups was conducted using Partial Least Squares Discriminant Analysis (PLS-DA) and Orthogonal Partial Least Squares Discriminant Analysis (OPLS-DA).

### Statistical analysis

Clinical data were analyzed using SPSS 26.0. Categorical variables were compared using the Chi-square test or Fisher’s exact test, chosen based on sample size and expected frequency for more reliable results. Continuous variables were compared using the t-test. All tests were two-sided, and a *p*-value <0.05 was considered statistically significant.

## Results

### Patient clinical characteristics

From October 2022 to October 2023, 70 lung cancer patients and 25 patients with benign lung diseases were enrolled from Tianjin Cancer Institute & Hospital and Tianjin Chest Hospital. The baseline characteristics of these groups are presented in Table 1. The lesions in the control group included interstitial lung disease and lesions appearing as nodules or tumor-like masses on imaging, which require differentiation from lung cancer, such as sarcoidosis, hamartoma, inflammatory nodules, and granulomas. Chronic bronchitis and COPD were also noted in the control group. Both groups were comparable in terms of age, sex, and smoking history.

**Table 1:** Baseline characteristics of patients.

Variable	Lung cancer group (*N* = 70)	Control group (*N* = 25)	*P*
Age(year)	63.2(±7.2)	61(±8.4)	0.588
Sex			0.404
Male	51 (72.9%)	16 (64%)	
Female	19 (27.1%)	9 (36%)	
Smoking status			0.09
Never	47 (67.1%)	12 (48%)	
Former/current	23 (32.9%)	13 (52%)	
Histology		–	
Adenocarcinoma	31 (44.3%)		
Squamous	27 (38.6%)		
Small cell carcinoma	12 (17.1%)		
Stage			
I-II	10 (14.3%)		
III-IV	50 (71.4%)		
Unknown	10 (14.3%)		
Benign lung diseases	–		
Interstitial lung disease		7 (28%)	
Sarcoidosis/hamartoma/inflammatory nodules/granulomas		12 (48%)	
Chronic bronchitis/COPD		5 (20%)	
Sleep apnea hypopnea syndrome		1 (4%)	

### The BALF microbiome in lung cancer and benign lung diseases

**Figure 2:**
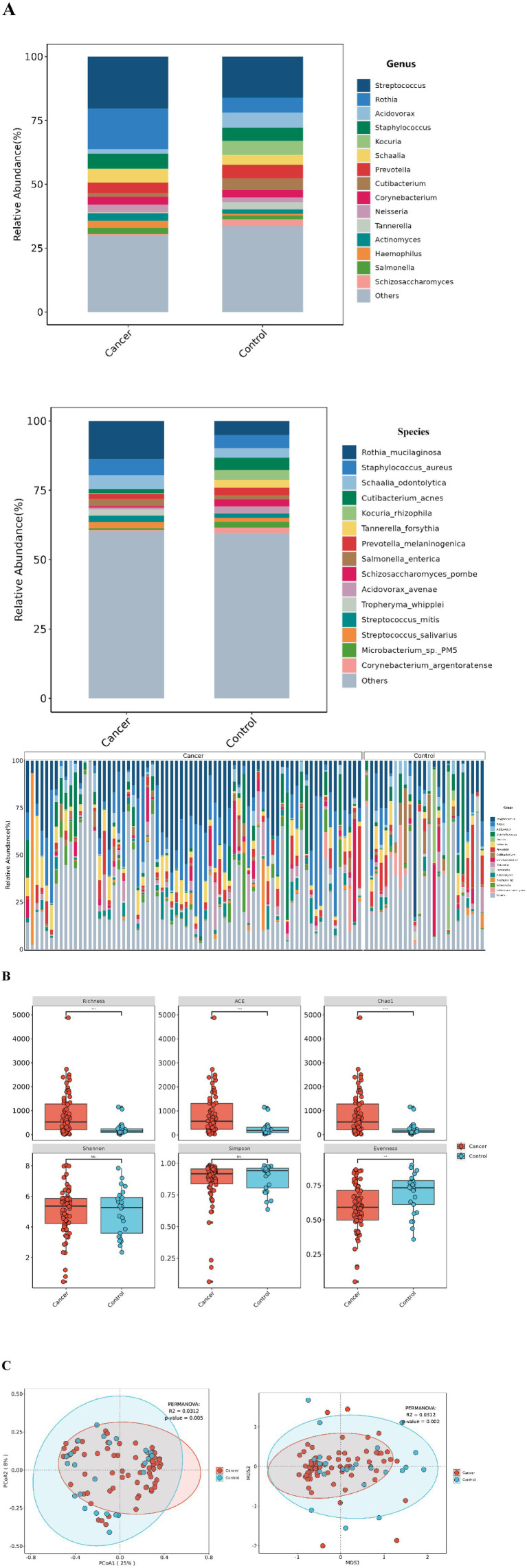
Lung microbiota composition in lung cancer patients and control group. **(A)** Microbial composition at the genus/species level for each group and distribution of the top 15 species (genus) in single-sample. **(B)** *α*-diversity between the two groups under different measurement indices, including: Shannon, Simpson, Richness, ACE, Chao1, and Evenness. ***p < 0.001; **p < 0.01; ns, no significant difference. **(C)** β-diversity between the lung cancer group and the control group based on PCoA and NMDS analyses.

Metagenomic sequencing was used to identify microbial species at the genus and species levels. At the genus level, 1,678 genera were detected, including various viruses and archaea. However, due to their low abundance, they were not further analyzed. Among the top 15 most abundant bacterial genera, similar genera were observed in both groups, although their proportions varied. In the lung cancer group, dominant genera included *Streptococcus* (20.27%), *Rothia* (15.88%), *Staphylococcus* (5.78%), *Schaalia* (5.44%), *Prevotella* (4.03%), *Corynebacterium* (3.10%), and *Neisseria* (3.03%). In the control group, dominant genera were *Streptococcus* (16.23%), *Rothia* (5.66%), *Acidovorax* (5.99%), *Kocuria* (5.56%), *Prevotella* (5.41%), *Cutibacterium* (4.51%), and *Schaalia* (3.73%). At the species level, 5,748 species were identified, with differences in species abundance between the groups. The most abundant species in the lung cancer group were *Rothia mucilaginosa* (13.86%), *Staphylococcus aureus* (5.72%), and *Schaalia odontolytica* (4.98%). In the control group, the most abundant were *Rothia mucilaginosa* (5.13%), *Staphylococcus aureus* (4.65%), *Cutibacterium acnes* (4.50%), *Kocuria rhizophila* (3.55%), and *Schaalia odontolytica* (3.46%) (Figure 2A).

### Decreased *β*-diversity in the lung cancer group

While no significant differences were observed in the Shannon and Simpson indices for *α*-diversity, significant differences were noted in the Richness, ACE, Chao1, and Evenness indices (Figure 2B). For β-diversity, PCoA and NMDS analyses based on the Bray-Curtis distance demonstrated clear separation between the groups, with the lung cancer group showing decreased β-diversity, statistically significant (*p* = 0.05, *R*^2^ = 0.0312) (Figure 2C).

### Differential BALF microbiota between lung cancer group and control group

**Figure 3:**
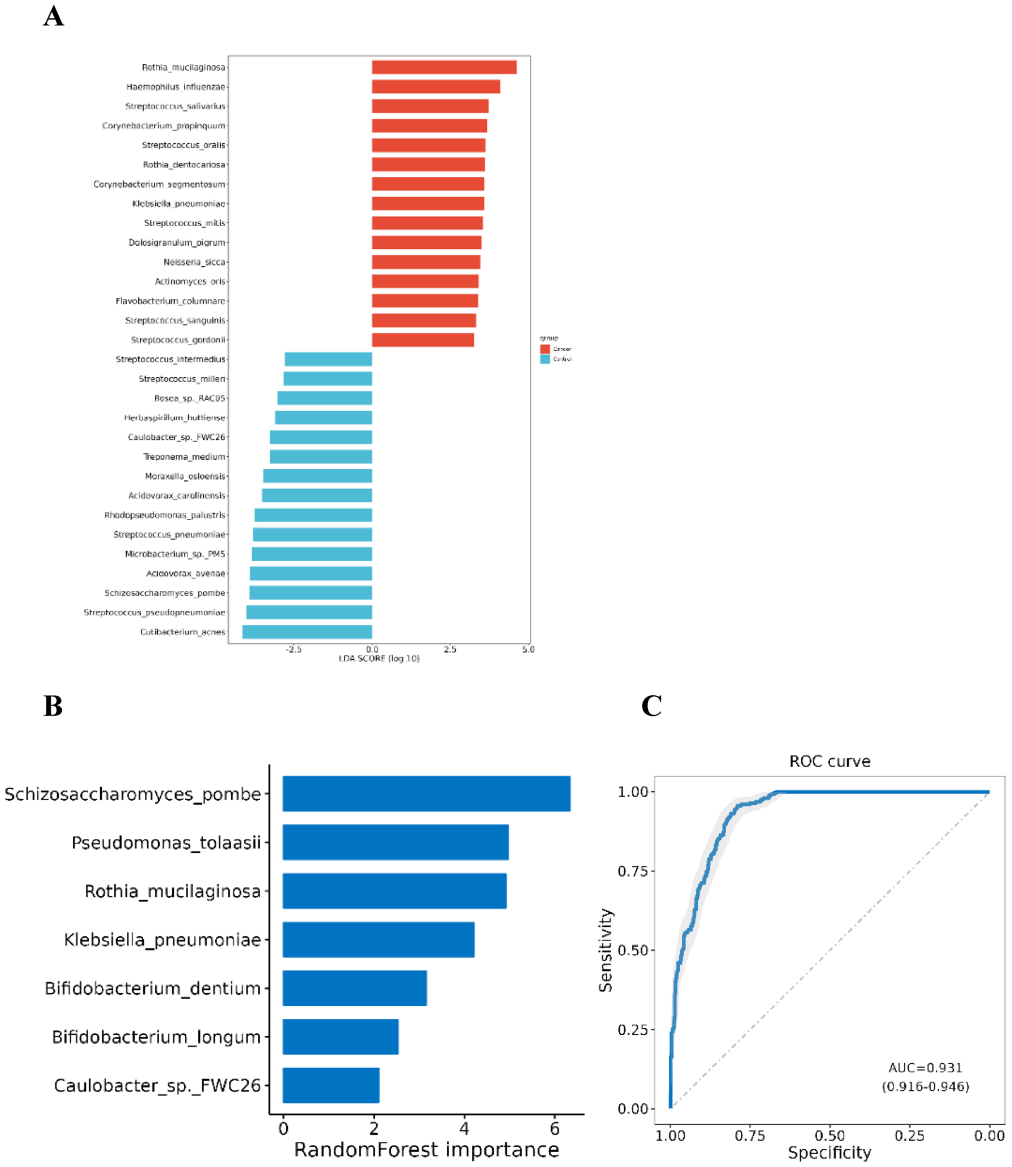
Differential microbial species between lung cancer group and control group. **(A)** Differential taxa at the species level identified by LEfSe analysis (Only species with a differential species count greater than 15 and in each group LDA score Top 15 are listed); **(B)** Species selected in the diagnostic model. **(C)** ROC curve for the diagnostic model.

LEfSe analysis was employed to detect microbes with significant differences at the species level between lung cancer and benign lung lesions. The analysis revealed that 78 species were significantly more abundant in the lung cancer group, whereas 26 species were significantly more abundant in the control group (Table 2, Supplementary Table 2, and Figure 3A).

**Table 2:** List of different microbial species in lung cancer group and control group by LEfSe analysis (LDA > 2.5).

Names	Log_value	Group	LDAscores	P_value
*Rothia_mucilaginosa*	5.14179	Cancer	4.622674	0.006578
*Cutibacterium_acnes*	4.65294	Control	4.16151	0.004059
*Haemophilus_influenzae*	4.278823	Cancer	4.092966	0.001062
*Streptococcus_pseudopneumoniae*	4.284667	Control	4.028614	0.023465
*Schizosaccharomyces_pombe*	4.404974	Control	3.924897	1.32E-07
*Streptococcus_pneumoniae*	4.232271	Control	3.800535	0.036392
*Streptococcus_salivarius*	4.351618	Cancer	3.724657	0.006133
*Streptococcus_oralis*	4.289609	Cancer	3.625046	0.021415
*Klebsiella_pneumoniae*	3.975981	Cancer	3.578428	0.015686
*Streptococcus_mitis*	4.352475	Cancer	3.532528	0.009683
*Streptococcus_sanguinis*	3.988466	Cancer	3.320127	0.004105
*Caulobacter_sp._FWC26*	3.650762	Control	3.264651	0.004005
*Streptococcus_gordonii*	3.759628	Cancer	3.258948	0.002572
*Streptococcus_australis*	3.674911	Cancer	3.071044	0.032637
*Veillonella_parvula*	3.646354	Cancer	3.000128	0.03438
*Pseudomonas_tolaasii*	3.28073	Cancer	2.867323	8.62E-06
*Streptococcus_milleri*	3.319731	Control	2.833595	0.041865
*Streptococcus_vestibularis*	3.224797	Cancer	2.824132	0.034245
*Streptococcus_intermedius*	3.338673	Control	2.782419	0.041813
*Streptococcus_sp._NPS_308*	3.329776	Cancer	2.71484	0.005176
*Streptococcus_sp._FDAARGOS_192*	3.146276	Cancer	2.691077	0.004757
*Bifidobacterium_longum*	3.056645	Cancer	2.646199	4.59E-05
*Neisseria_sp._oral_taxon_014*	3.002919	Control	2.616063	0.016194
*Bifidobacterium_dentium*	2.910455	Cancer	2.586571	1.75E-05

Using the significantly different species between the two groups, the Sequential Forward Feature Selection algorithm and Random Forest Classifier were utilized to select a subset of species that maximally distinguished the two groups. A diagnostic model was developed based on seven species: *Pseudomonas tolaasii*, *Rothia mucilaginosa*, *Klebsiella pneumoniae*, *Bifidobacterium dentium*, *Bifidobacterium longum*, *Schizosaccharomyces pombe*, and *Caulobacter* sp. *FWC26*. The first five species were significantly enriched in the lung cancer group, while the latter two were more prevalent in the control group. The AUC of this model for distinguishing lung cancer from benign lung lesions was 0.931 (95% CI: 0.916–0.946), indicating significant differences in BALF microbiota between lung cancer and benign lesions, potentially serving as microbial biomarkers for lung cancer (Figures 3B,C).

### Microbial-related gene function prediction

**Figure 4:**
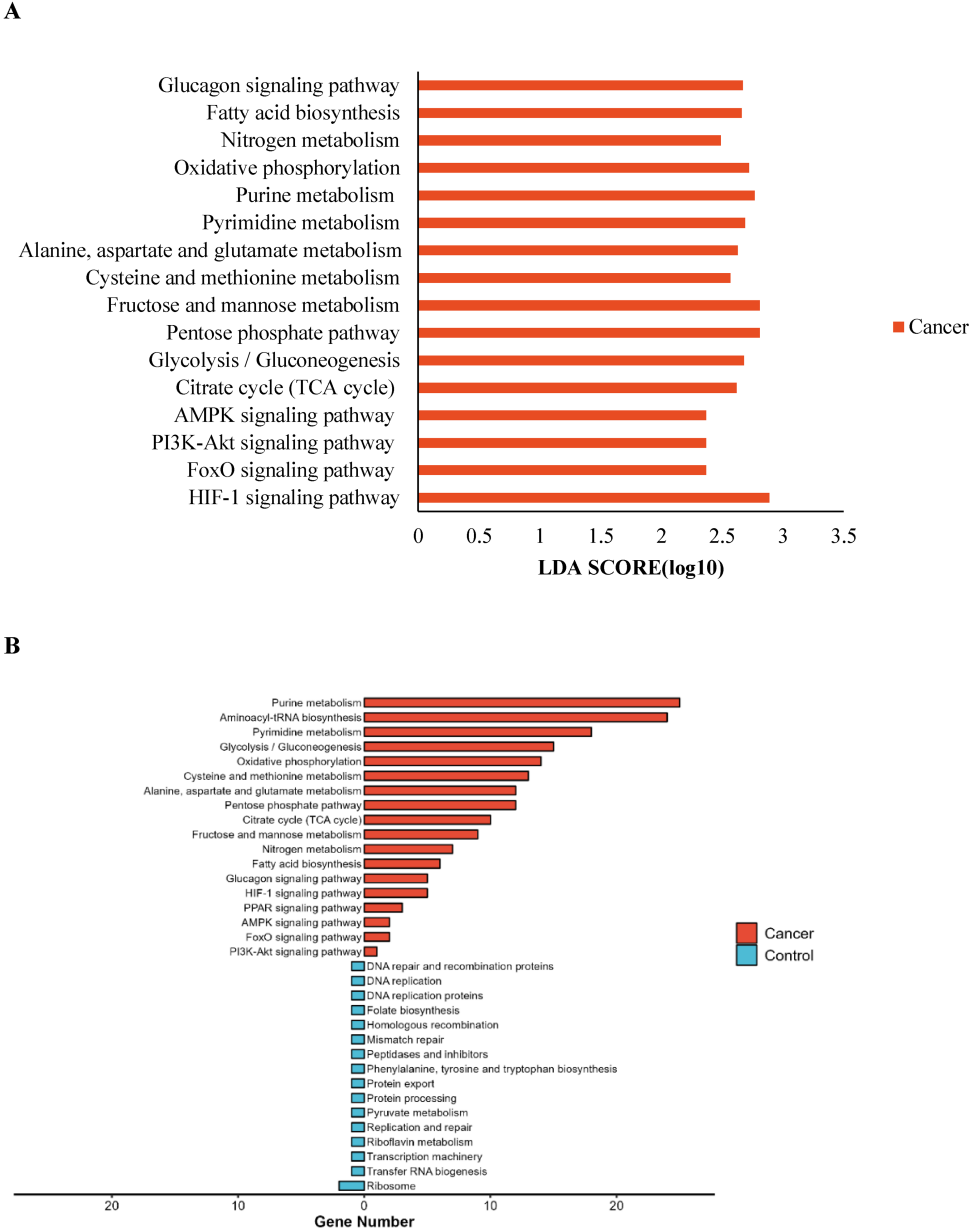
Prediction of lung microbiota function. **(A)** The impact of differentially enriched KEGG pathways in lung cancer group was evaluated through the LDA score; **(B)** Number of genes annotated in the differential KEGG pathways.

HUMAnN2 and LEfSe were used to analyze microbial pathways. KEGG pathway analysis revealed significant upregulation in the lung cancer group of pathways including signal transduction (e.g., HIF-1 signaling pathway, PI3K-Akt signaling pathway), carbohydrate metabolism (e.g., Citrate cycle [TCA cycle], Glycolysis/Gluconeogenesis), nucleotide metabolism (Pyrimidine metabolism, Purine metabolism), amino acid metabolism (Cysteine and methionine metabolism, Alanine, aspartate, and glutamate metabolism), and energy metabolism (Oxidative phosphorylation, Nitrogen metabolism). This upregulation suggests that catabolic and anabolic activities are more active in lung cancer patients, and tumor growth-related signaling pathways are significantly enhanced, consistent with the hypercatabolic state and malignant characteristics of lung cancer (Figures 4A,B).

### BALF metabolomics

#### Metabolite composition and distribution

**Figure 5:**
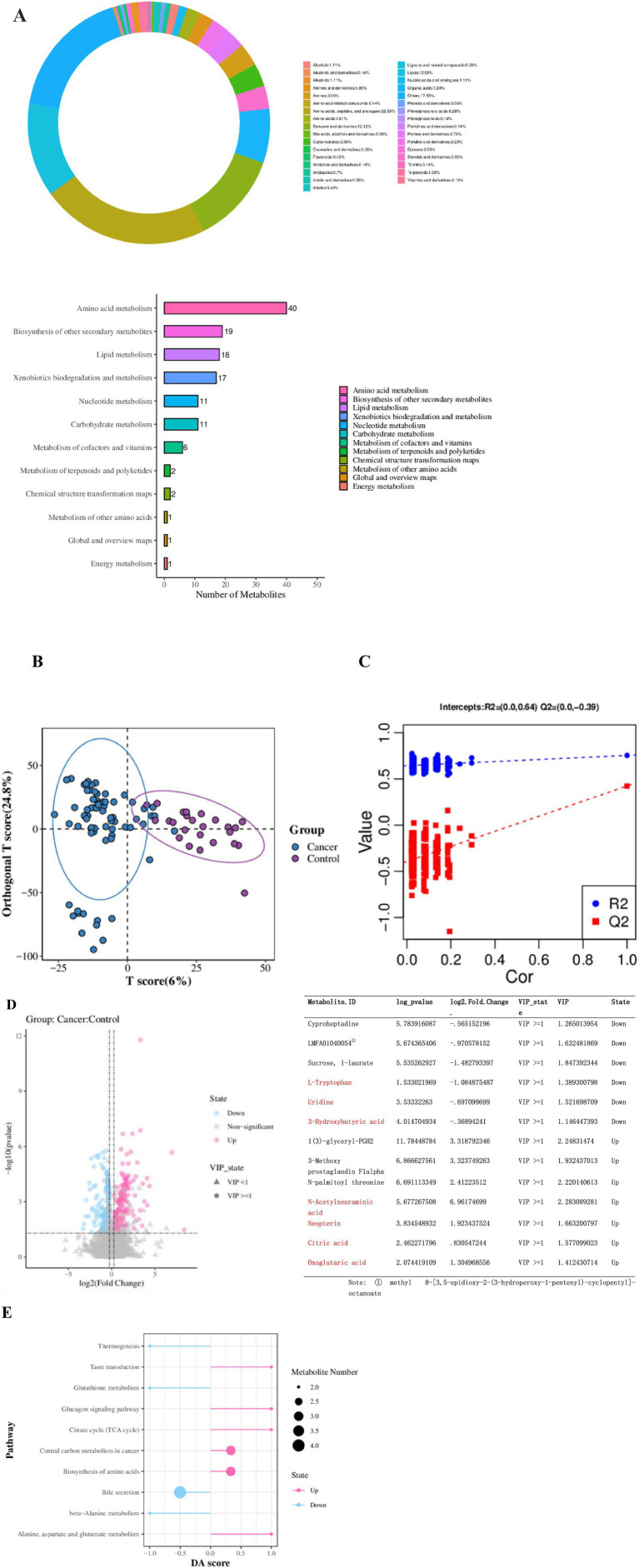
Metabolite Classification and Function Prediction. **(A)** Metabolite classification of the two groups. **(B)** Score diagram of the OPLS-DA analysis model. **(C)** Replacement test chart of the OPLS-DA analysis model. **(D)** Volcano plot of differential metabolites (Fold Change calculated for each metabolite in each group and significance tested using Student’s *t*-test: VIP ≥ 1 in the OPLS-DA model, Fold Change ≥ 1.2 or ≤ 0.83, *p*-value < 0.05). The list shows the top three metabolites that are significantly upregulated or downregulated, as well as the metabolites we are concerned about. **(E)** Metabolic KEGG pathway enrichment analysis.

A total of 3,653 metabolites were detected in the 95 BALF samples from both groups. Quality control indicators confirmed the stability, reproducibility, and quality of detection (Supplementary Figures 1A–D). Among these, 1,249 metabolites were successfully identified. The four primary categories, comprising over 50% of the total, included Amino acids, peptides, and analogs (22.56%), Lipids (12.53%), Benzene and derivatives (12.12%), and Organic acids (7.24%). Metabolites were categorized according to the KEGG super pathway, revealing participation primarily in Amino acid metabolism, Biosynthesis of other secondary metabolites, Lipid metabolism, Xenobiotics biodegradation and metabolism, Nucleotide metabolism, and Carbohydrate metabolism (Figure 5A).

#### Significant differences in metabolites between lung cancer group and control group

The OPLS-DA model was utilized to analyze differences in metabolites between the groups. A replacement test confirmed the model was not overfitted and performed effectively (Figures 5B,C). A total of 261 significantly different metabolites were identified, with 144 metabolites significantly upregulated and 117 metabolites significantly downregulated in the lung cancer group. Notable metabolites increased in the lung cancer group included Citric acid, *N*-Acetylneuraminic acid, Oxoglutaric acid, and Neopterin, while those decreased included L-Tryptophan, Uridine, and 3-Hydroxybutyric acid (Figure 5D).

Significantly upregulated KEGG pathways among the top 10 metabolic pathways with the smallest *p*-values in the lung cancer group included: Alanine, aspartate, and glutamate metabolism, Biosynthesis of amino acids, Central carbon metabolism in cancer, TCA cycle, and Glucagon signaling pathway. Significantly downregulated pathways included: beta-Alanine metabolism, Bile secretion, Glutathione metabolism, and Thermogenesis (Figure 5E).

#### Changes in BALF metabolites are closely related to lung microbiota in lung cancer

**Figure 6:**
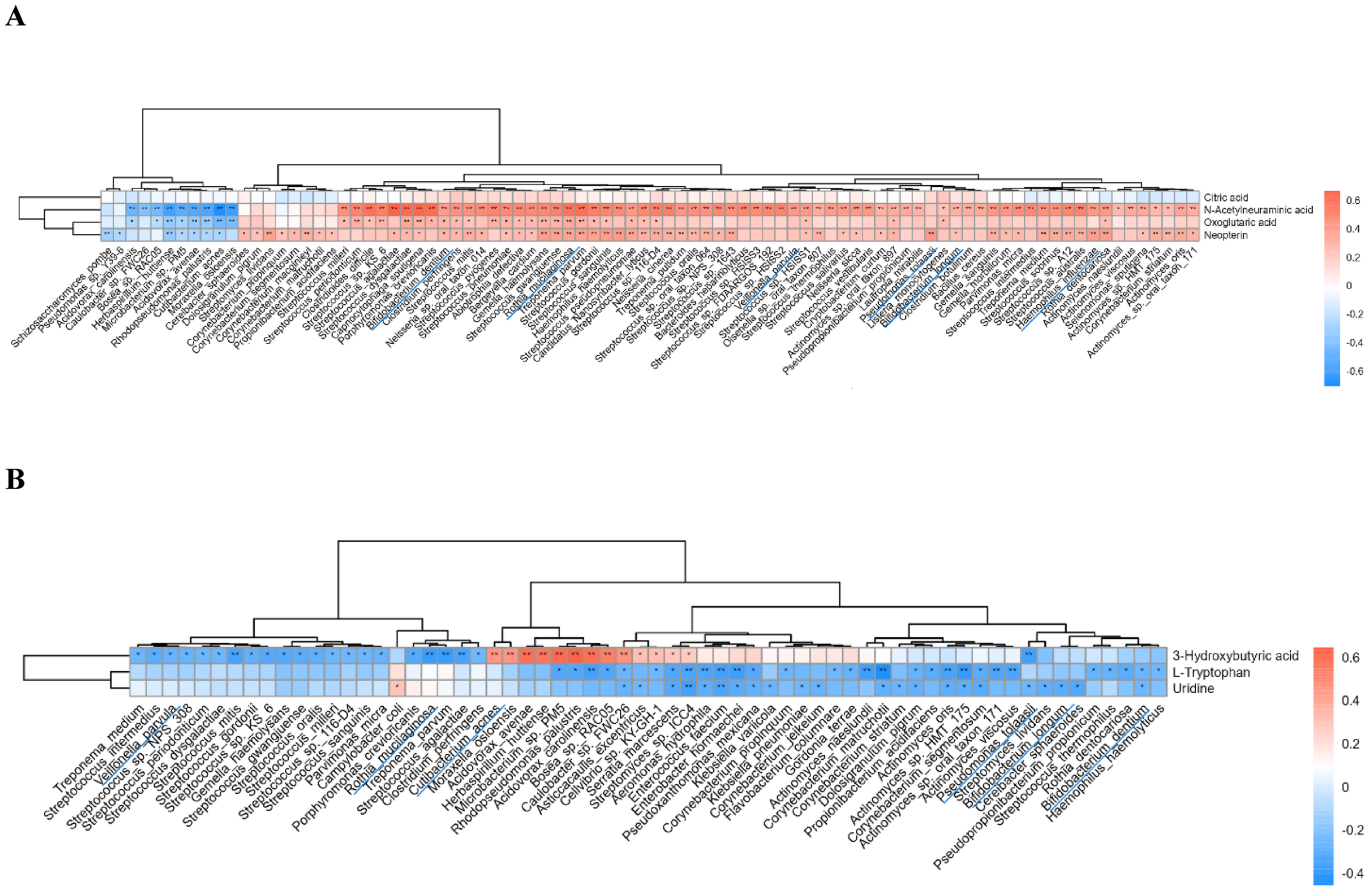
Correlation analysis of differential metabolites and microbial species. **(A)** Correlation of upregulated metabolites in the lung cancer group with microbiota; **(B)** Correlation of downregulated metabolites in the lung cancer group with microbiota. **p < 0.01, *p < 0.05.

Combined analysis of differential metabolites and microbial species revealed correlations between several species and metabolite changes. Species such as *Streptococcus*, which were highly expressed in the lung cancer group, and *Pseudomonas tolaasii*, *Rothia mucilaginosa*, *Bifidobacterium dentium*, *Bifidobacterium longum*, *Haemophilus influenzae*, and *Veillonella parvula* were positively correlated with the upregulated metabolites. The same species, except *Haemophilus influenzae*, showed negative correlations with the downregulated metabolites. Additionally, *Cutibacterium acnes* was significantly positively correlated with 3-Hydroxybutyric acid (Figures 6A,B).

## Discussion

In this study, the relationship between lower respiratory tract microbiota and lung cancer, as well as benign lung lesions, was explored using BALF as the sample source. It was found that microbial richness in lung cancer patients was higher than in those with benign lung diseases, and the Evenness index was significantly lower. However, no significant differences were observed in the Shannon and Simpson indices, which are commonly used to measure microbial *α*-diversity. Previous studies have reported inconsistent findings regarding α-diversity between lung cancer patients and healthy or benign controls. For example, Cheng reported significantly lower α-diversity richness in lung cancer patients using 16 s rRNA sequencing (Cheng et al., 2024), while Jin noted a high correlation between lung tumor burden and local bacterial abundance (Jin et al., 2019). The observed higher microbial richness in our study’s lung cancer patients could be related to the higher proportion of stage III-IV patients. Conversely, Kim found that the Shannon and Evenness indices were significantly higher in lung cancer patients (Kim et al., 2024), yet other studies reported no differences in α-diversity between benign and malignant lung diseases (Jin et al., 2019; Tsay et al., 2021; Liu et al., 2022).

α-Diversity in microbial research is characterized by indices such as Richness, ACE, Chao1, Shannon, Simpson, and Evenness. It was observed that not all studies comprehensively presented differences across these indices. While these indices all reflect species diversity within a community, they emphasize different aspects, and comparisons across studies using various indices may not always be appropriate. Factors such as sample size, sampling methods, and sample storage time might also contribute to differences in microbial communities.

*β*-Diversity in lung cancer patients was found to be significantly lower than in those with benign lung disease. Lower β-diversity suggests a more consistent microbial composition in the BALF of lung cancer patients (Jin et al., 2019; Cheng et al., 2024; Hosgood et al., 2019; Lu et al., 2021), indicating that changes in lower respiratory tract microbiota are crucial in the development and progression of lung cancer.

The study also analyzed differences in BALF microbial species between lung cancer and benign lung disease. *Streptococcus* species were significantly enriched in lung cancer patients, aligning with previous findings (Table 2) (Jin et al., 2019; Kim et al., 2024). Tsay confirmed that exposure to *Prevotella, Streptococcus,* and *Veillonella* upregulated ERK and PI3K signaling pathways in lung cancer (Tsay et al., 2021). A significant increase in *Haemophilus influenzae* was observed in lung cancer patients. King reported that this bacterium’s outer membrane proteins, like P2 and P6, activate innate immunity, with P6 stimulating macrophages to produce interleukin-8 (IL-8) and tumor necrosis factor-*α* (TNF-α). Furthermore, non-encapsulated strains (NTHi) of *Haemophilus influenzae* have been shown to upregulate nuclear factor-κB (NF-κB) (King, 2012), a pathway crucial for cell proliferation and survival. IL-8 has been associated with tumor growth and metastasis through mechanisms like epithelial-mesenchymal transition and angiogenesis (Yuen et al., 2020). The NF-κB pathway is key in promoting cell proliferation, survival, and metastasis (Taniguchi and Karin, 2018). Additionally, significant enrichment of *Veillonella parvula* was noted in lung cancer patients (Jin et al., 2019; Tsay et al., 2021; Segal et al., 2016), with previous studies showing its association with Th17 lymphocyte-dependent local inflammatory responses (Tsay et al., 2021), where Th17 lymphocytes secrete IL-17, involved in tumor immunity and angiogenesis (Onishi and Gaffen, 2010; Zhang et al., 2009).

Additionally, we observed significant enrichment of *Cutibacterium acnes* in the BALF of patients with benign lung diseases, which is among the most common bacteria on the skin. Lawson suggested that *Cutibacterium acnes* might be linked to prostate cancer (Lawson and Glenn, 2022). However, other studies have shown that the immunomodulatory activity of *Cutibacterium acnes* provides potential antitumor properties and has been utilized as a vaccine adjuvant (Simonart, 2013). Our study revealed a significant positive correlation between *Cutibacterium acnes* and the metabolite 3-hydroxybutyric acid, which may inhibit tumor growth. This indicates that the enrichment of *Cutibacterium acnes* in the BALF of benign lung disease patients may offer a protective antitumor effect.

Given the significant microbial differences between lung cancer and benign lung diseases, we constructed a prediction model that achieved an AUC value of 0.931, indicating robust performance in distinguishing between benign and malignant lung lesions. The model includes seven species: *Pseudomonas tolaasii*, *Rothia mucilaginosa*, *Klebsiella pneumoniae*, *Bifidobacterium dentium*, *Bifidobacterium longum*, *Schizosaccharomyces pombe*, and *Caulobacter* sp. *FWC26*. Literature has documented the enrichment of *Pseudomonas* and *Rothia* in lung cancer patients (Roy et al., 2022; Jin et al., 2019; Cheng et al., 2024). However, as many previous studies relied on 16 s rRNA sequencing, which primarily identifies microbiota at the genus level, the role of *Pseudomonas tolaasii* and *Rothia mucilaginosa* in lung cancer pathogenesis remains uncertain. *Klebsiella pneumoniae* is a prevalent opportunistic pathogen in lung cancer patients. Greathouse discovered that five genera, including *Klebsiella*, were closely associated with mutations in the tumor protein p53 (TP53) in patients with lung squamous cell carcinoma (Greathouse et al., 2018). Research on *Bifidobacterium* has mainly focused on the *gastrointestinal tract*, emphasizing its beneficial role and its potential to suppress tumor growth (Ghoddusi and Tamime, 2014; Lee et al., 2021). Our study suggests that the presence of *Bifidobacterium* in the respiratory tract might promote tumor growth through metabolite regulation. However, the potential for sample contamination cannot be completely dismissed. It is widely recognized that daily consumption of yogurt products often includes various intestinal probiotics. Although we attempted to exclude individuals taking oral probiotics during the initial enrollment phase, yogurt products are commonly consumed as part of daily diets.

We found that *Pseudomonas tolaasii*, *Rothia mucilaginosa*, *Bifidobacterium dentium*, and *Bifidobacterium longum* were either positively or significantly positively correlated with four upregulated metabolites in the lung cancer group. Conversely, these species were negatively or significantly negatively correlated with downregulated metabolites. These metabolite changes are linked with tumor development (which will be discussed in the subsequent section), suggesting that these microbial species may enhance tumor growth through metabolites.

The components of BALF primarily consist of cells and soluble substances, mostly originating from plasma, with a smaller portion produced locally. However, changes in BALF composition often do not correspond with those in blood, necessitating an analysis of BALF components. In our study, compared to the benign lung disease group, the levels of citric acid, N-acetylneuraminic acid, oxoglutaric acid, and neopterin were markedly increased in the BALF of lung cancer patients, while L-tryptophan, uridine, and 3-hydroxybutyric acid were markedly decreased. Citric acid and oxoglutaric acid, key intermediate metabolites in the TCA cycle, are involved in the metabolism of carbohydrates, fats, and proteins. An increase in citric acid suggests elevated metabolic activity in tumors. Elevated levels of these two substances in BALF indicate that their concentrations may align with those in plasma. Neopterin, a metabolite of guanosine triphosphate produced by *γ*-interferon-stimulated monocytes and macrophages, can remain stable in body fluids such as blood, pleural, and ascites for a long time. Elevated neopterin, observed in various malignancies, is associated with poor prognosis (Melichar et al., 2017). In our study, significantly increased levels of neopterin in the BALF of lung cancer patients suggest that its levels in BALF may correspond to those in plasma and other body fluids. *N*-acetylneuraminic acid, a component of cell surfaces, is the most widely distributed form of sialic acid in nature. *N*-acetylneuraminic acid is notably increased on tumor cell surfaces and in plasma (Fischer and Egg, 1990), leading to an increase in negative charge on the cell surface and enhanced repulsive forces between cells, thereby reducing intercellular adhesion. This process facilitates the detachment of tumor cells from the primary tumor, creating conditions for invasion and metastasis. Researchers have found that *N*-acetylneuraminic acid in urine are associated with lung cancer risk and may increase with tumor size, making it a potential auxiliary tool for early lung cancer diagnosis (Zhang et al., 2021). In our study, *N*-acetylneuraminic acid levels in BALF were also significantly elevated, with an AUC of 0.727 when used alone in the diagnostic model, indicating its potential utility in lung cancer diagnosis. Previous studies have reported increased levels of L-tryptophan, uridine, and 3-hydroxybu tyric acid in the plasma of patients with malignant tumors (Hilvo et al., 2016; Badawy, 2022; Vidman et al., 2023; Yang et al., 2024). Tumor tissues utilize tryptophan and its metabolites to promote growth and evade host defense (Badawy, 2022). Uridine metabolism plays a critical role in tumorigenesis by providing uridine diphosphate *N*-acetylglucosamine (UDP-GlcNAc) (Dmitrieva-Posocco et al., 2022). Conversely, 3-hydroxybutyric acid has been demonstrated to inhibit the growth of colon cancer (Dmitrieva-Posocco et al., 2022). In our study, these three metabolites were significantly decreased in the BALF of lung cancer patients. Additionally, we discovered that *Pseudomonas tolaasii*, *Rothia mucilaginosa*, *Bifidobacterium dentium*, *Bifidobacterium longum*, and *Veillonella parvula*, significantly enriched in the lung cancer group, were negatively or significantly negatively correlated with these downregulated metabolites. This suggests that the discrepancy between plasma and BALF levels of these metabolites may be influenced by the activity of airway microbiota.

Our study also has several limitations. First, the sample size was relatively small (*n* = 70 vs. *n* = 25), with a significant disparity in the number of cases between the two groups, which can affect model performance estimates and statistical comparisons. Additionally, no external model was used for further validation. Second, the collection of BALF makes it difficult to completely avoid contamination from oral flora, and the influence of diet is also challenging to eliminate entirely. Furthermore, the microbial environment in the bronchus where the tumor is located may differ from that in non-tumor lesion areas. However, the bronchoalveolar lavage process may potentially mix contents from multiple bronchi.

## Conclusion

Microbial metagenomics and metabolomics were utilized to analyze the differences in microorganisms and metabolites in BALF between lung cancer patients and those with benign lung diseases. Significant differences were identified in the BALF microbiota of lung cancer patients compared to those with benign lung diseases. The microbial model in the BALF of lung cancer patients could serve as a biomarker for diagnosis. Moreover, the significantly altered microorganisms and metabolites in the BALF of lung cancer patients are linked with tumorigenesis and progression.

## Data Availability

The data presented in the study are deposited in the Genome Sequence Archive (Genomics, Proteomics & Bioinformatics 2025) in National Genomics Data Center (Nucleic Acids Res 2025), China National Center for Bioinformation / Beijing Institute of Genomics, Chinese Academy of Sciences repository, accession number (GSA) CRA031764.
