# Supplementary material for: Microbiome-metabolome dysbiosis of bronchoalveolar lavage fluid of lung cancer patients
Source: Front Microbiol. 2025 Nov 12;16:1669172. doi: 10.3389/fmicb.2025.1669172 (PMC12647101; doi:10.3389/fmicb.2025.1669172)
Supplement: Supplementary file 1 [file Data_Sheet_1.docx]

**Microbiome-Metabolome Dysbiosis of Bronchoalveolar Lavage Fluid of Lung Cancer Patients**

Supplementary Table 1: Sequencing Data Statistics

Supplementary Table 2. List of different microbial species in lung cancer group and control group by LEfSe analysis (LDA＞2.5)

| **Names** | **Log_value** | **group** | **LDAscores** | **P_value** |
| --- | --- | --- | --- | --- |
| *Rothia_mucilaginosa* | 5.14179 | Cancer | 4.622674 | 0.006578 |
| *Cutibacterium_acnes* | 4.65294 | Control | 4.16151 | 0.004059 |
| *Haemophilus_influenzae* | 4.278823 | Cancer | 4.092966 | 0.001062 |
| *Streptococcus_pseudopneumoniae* | 4.284667 | Control | 4.028614 | 0.023465 |
| *Schizosaccharomyces_pombe* | 4.404974 | Control | 3.924897 | 1.32E-07 |
| *Acidovorax_avenae* | 4.373669 | Control | 3.904867 | 0.00677 |
| *Microbacterium_sp._PM5* | 4.330059 | Control | 3.855759 | 0.022731 |
| *Streptococcus_pneumoniae* | 4.232271 | Control | 3.800535 | 0.036392 |
| *Rhodopseudomonas_palustris* | 4.280203 | Control | 3.755848 | 0.011699 |
| *Streptococcus_salivarius* | 4.351618 | Cancer | 3.724657 | 0.006133 |
| *Corynebacterium_propinquum* | 4.15292 | Cancer | 3.676594 | 0.012099 |
| *Streptococcus_oralis* | 4.289609 | Cancer | 3.625046 | 0.021415 |
| *Rothia_dentocariosa* | 4.018076 | Cancer | 3.608246 | 0.006216 |
| *Corynebacterium_segmentosum* | 3.868096 | Cancer | 3.580247 | 0.015274 |
| *Klebsiella_pneumoniae* | 3.975981 | Cancer | 3.578428 | 0.015686 |
| *Streptococcus_mitis* | 4.352475 | Cancer | 3.532528 | 0.009683 |
| *Acidovorax_carolinensis* | 3.916549 | Control | 3.517185 | 0.029155 |
| *Dolosigranulum_pigrum* | 3.876306 | Cancer | 3.498889 | 0.003004 |
| *Moraxella_osloensis* | 4.020472 | Control | 3.476997 | 0.025085 |
| *Neisseria_sicca* | 3.891275 | Cancer | 3.460202 | 0.040312 |
| *Actinomyces_oris* | 3.900151 | Cancer | 3.416472 | 0.003858 |
| *Flavobacterium_columnare* | 3.765616 | Cancer | 3.382774 | 0.000121 |
| *Streptococcus_sanguinis* | 3.988466 | Cancer | 3.320127 | 0.004105 |
| *Treponema_medium* | 3.562121 | Control | 3.269738 | 0.047736 |
| *Caulobacter_sp._FWC26* | 3.650762 | Control | 3.264651 | 0.004005 |
| *Streptococcus_gordonii* | 3.759628 | Cancer | 3.258948 | 0.002572 |
| *Pseudopropionibacterium_propionicum* | 3.608299 | Cancer | 3.257894 | 0.049998 |
| *Streptococcus_sp._A12* | 3.566736 | Cancer | 3.11856 | 0.043019 |
| *Lautropia_mirabilis* | 3.439015 | Cancer | 3.11076 | 0.014687 |
| *Gemella_haemolysans* | 3.610243 | Cancer | 3.103985 | 0.003754 |
| *Herbaspirillum_huttiense* | 3.665003 | Control | 3.103858 | 0.001165 |
| *Actinomyces_sp._HMT_175* | 3.543372 | Cancer | 3.086443 | 0.008154 |
| *Streptococcus_australis* | 3.674911 | Cancer | 3.071044 | 0.032637 |
| *Klebsiella_variicola* | 3.583683 | Cancer | 3.054639 | 0.047058 |
| *Bosea_sp._RAC05* | 3.420453 | Control | 3.021419 | 0.020473 |
| *Veillonella_parvula* | 3.646354 | Cancer | 3.000128 | 0.03438 |
| *Gemella_morbillorum* | 3.361374 | Cancer | 2.984728 | 0.02144 |
| *Abiotrophia_defectiva* | 3.463507 | Cancer | 2.948109 | 0.015315 |
| *Bacteroides_heparinolyticus* | 3.270885 | Cancer | 2.92507 | 0.02374 |
| *Parvimonas_micra* | 3.40372 | Cancer | 2.916409 | 0.041542 |
| *Pseudomonas_tolaasii* | 3.28073 | Cancer | 2.867323 | 8.62E-06 |
| *Streptococcus_milleri* | 3.319731 | Control | 2.833595 | 0.041865 |
| *Streptococcus_vestibularis* | 3.224797 | Cancer | 2.824132 | 0.034245 |
| *Enterobacter_hormaechei* | 3.148936 | Cancer | 2.809455 | 0.002979 |
| *Gemella_sanguinis* | 3.394559 | Cancer | 2.783841 | 0.009618 |
| *Streptococcus_intermedius* | 3.338673 | Control | 2.782419 | 0.041813 |
| *Corynebacterium_striatum* | 2.933766 | Cancer | 2.768703 | 0.001519 |
| *Streptococcus_gwangjuense* | 3.382887 | Cancer | 2.75138 | 0.006399 |
| *Candidatus_Nanosynbacter_lyticus* | 3.22968 | Cancer | 2.744848 | 0.006175 |
| *Qipengyuania_flava* | 2.992993 | Cancer | 2.737102 | 0.036484 |
| *Streptococcus_sp._NPS_308* | 3.329776 | Cancer | 2.71484 | 0.005176 |
| *Streptococcus_sp._FDAARGOS_192* | 3.146276 | Cancer | 2.691077 | 0.004757 |
| *Actinomyces_naeslundii* | 3.301161 | Cancer | 2.690245 | 0.014201 |
| *Streptococcus_thermophilus* | 3.150927 | Cancer | 2.689823 | 0.008688 |
| *Streptomyces_lividans* | 3.224522 | Cancer | 2.66985 | 0.002189 |
| *Corynebacterium_macginleyi* | 2.944859 | Cancer | 2.659663 | 0.000626 |
| *Streptococcus_sp._HSISS1* | 3.01909 | Cancer | 2.659136 | 0.005047 |
| *Capnocytophaga_sputigena* | 2.995835 | Cancer | 2.656947 | 0.01297 |
| *Bifidobacterium_longum* | 3.056645 | Cancer | 2.646199 | 4.59E-05 |
| *Neisseria_sp._oral_taxon_014* | 3.002919 | Control | 2.616063 | 0.016194 |
| *Bifidobacterium_dentium* | 2.910455 | Cancer | 2.586571 | 1.75E-05 |
| *Actinomyces_sp._oral_taxon_171* | 3.212086 | Cancer | 2.585148 | 0.04565 |
| *Cereibacter_sphaeroides* | 3.000904 | Cancer | 2.52598 | 0.001082 |

A B


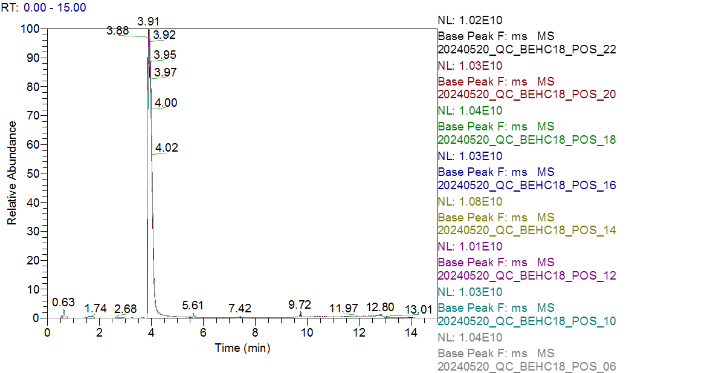

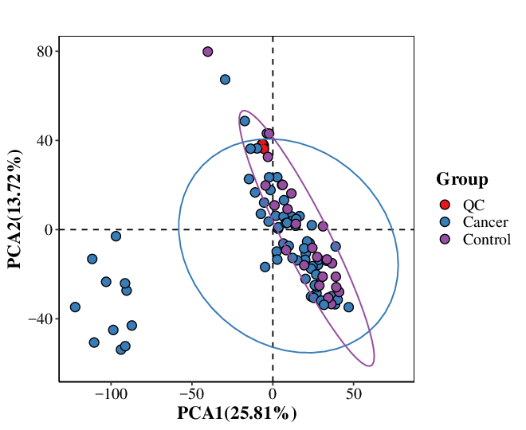


C


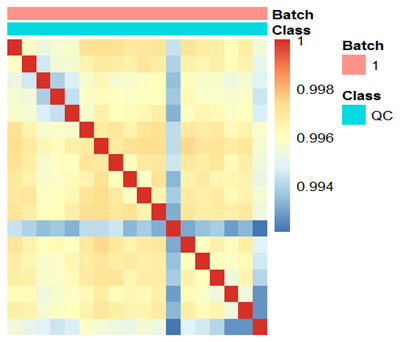


D


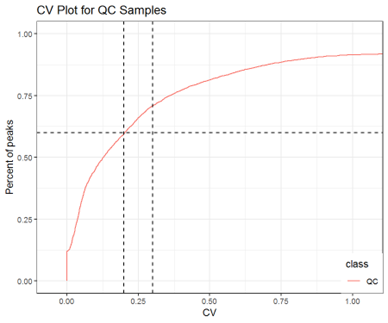


CV assessment table on QC sample

| **Num of Total** | **Num of CV≤30%** | **Ratio of CV≤30%** |
| --- | --- | --- |
| 5158 | 3653 | 0.71 |

Supplementary Figure 1.Quality Control Evaluation Metrics for QC Samples. (**A**) BPC Overlapping Spectrum of QC Samples. Note: Overlapping the BPC (base peak chromatograms) of all QC samples, and the result of good chromatograms overlap, little fluctuation of retention time and peak response intensity showed that the instrument is in good condition and the signal is stable in the whole process of sample detection and analysis. (**B**) PCA Score Graph of All Samples. NOTE: PC1 (25.81%) represents the first principal component and PC2 (13.72%) represents the second principal component. Percentage represents the interpretation rate of the principal component to the dataset and the ellipse represents a 95% confidence interval. The better the QC samples are gathered, the more stable the instrument is and the better the repeatability of the collected datais. (**C**) Correlation Heatmap on QC samples. The correlation between the colors of small squares increases from blue to red. (**D**) CV Distribution of Compounds in Each Group of Samples. In the figure, the two lines perpendicular to the X-axis are respectively 20% and 30% CV reference lines, and the lines parallel to the X-axis are 60% reference lines. Note: After select all QC samples from all samples, By calculating Coefficient of Variation (CV) of every Metabolite intensity in QC samples, Reputability of the QC sample could be detected. The higher the proportion of compounds with low CV value in QC samples is, the more stable the experimental data is. The proportion of compounds with CV value less than 0.3 in QC samples was higher than 60%,indicating that the quantity of experimental data were qualified.
